# The role of crude saliva and purified salivary mucins in the inhibition of the Human Immunodeficiency Virus type 1

**DOI:** 10.1186/1743-422X-9-177

**Published:** 2012-08-28

**Authors:** Julia Peacocke, Zoe Lotz, Corena de Beer, Paul Roux, Anwar S Mall

**Affiliations:** 1 Department of Surgery, Division of General Surgery, University of Cape Town, Observatory, Cape, 7925, South Africa; 2 Discipline of Medical Virology, University of Stellenbosch and National Health Laboratory Service, Tygerberg, South Africa; 3 Department of Paediatric Medicine, University of Cape Town, Observatory, Cape, 7925, South Africa

**Keywords:** Mucin, MUC5B, MUC7, HIV-1, Inhibition assay

## Abstract

**Background:**

Sub-Saharan Africa is the world’s worst HIV-AIDS affected region. More interventions to manage this pandemic are urgently required. Transmission of the virus through an exchange of saliva is rarely known to occur. This project sought to verify statistically previous findings in our laboratory, that crude saliva from uninfected individuals together with its purified mucin components inhibited HIV-1, whilst mucins from infected saliva did not show this inhibition, in an *in vitro* assay.

**Methods:**

**Saliva** was extracted in 4 M guanidinium hydrochloride and proteolytic inhibitors at pH 6.5, followed by the isolation of MUC5B and MUC7 by Sepharose 4B gel filtration and further purification of these mucins by density-gradient ultra-centrifugation in caesium chloride. Agarose gel electrophoresis, Western blotting and amino acid compositional analysis determined the size, purity and identity of the mucins. The inhibitory activity of crude saliva and purified MUC5B and MUC7, from HIV negative (n=20) and HIV positive (n=20) donors, was tested by their incubation with subtype C HIV-1 and subsequent infection of peripheral blood mononuclear cells (PBMCs). PCR was done on tandem repeat regions of MUC5B and MUC7 DNA to investigate whether any association existed between gene polymorphism and susceptibility to infection.

**Results:**

There was an inter-individual variation in the amounts of MUC5B and MUC7 in saliva. In contrast to previous studies, crude saliva and purified mucins from both HIV negative and HIV positive individuals inhibited the infection of HIV-1 in an *in vitro* assay. DNA analysis of the tandem repeat regions of MUC5B and MUC7 revealed no difference between groups.

**Conclusions:**

Crude saliva and its mucins, MUC5B and MUC7, from both uninfected controls and HIV positive individuals inhibited HIV-1 in an *in vitro* assay.

## Background

Human Immunodeficiency Virus – Acquired Immune Deficiency Syndrome (HIV-AIDS) is a highly prevalent disease within the Sub-Saharan region including South Africa [1]. The broad aim of this study was to investigate the role of salivary mucus and mucins (mucous glycoproteins) in HIV-AIDS. Sexual promiscuity and the secondary status of women in certain sectors of society increase the risk for HIV transmission in these populations [1].

In a definitive study from our laboratory by Habte *et al *[2], it was shown qualitatively that saliva from uninfected individuals together with its purified mucin components MUC5B and MUC7 inhibited the HI-virus in an *in vitro* assay. Habte *et al *[3] further showed that MUC5B and MUC7 from saliva of infected individuals did not inhibit the virus in an *in vitro* assay.

Both this study and the previous ones by Habte *et al *[2,3] are based on the assumption of the presence of infectious, viable HIV in the saliva [4]. Several researchers proposed the presence of a key macromolecular component in saliva that could play a significant role in preventing replication and subsequent infection of receptive cells by HIV-1 [2,5-7]. A study by Archibald *et al. *[8] investigated the *in vitro* inhibitory activities of saliva against the HIV-1 virus and found that whole saliva and specific glandular salivas, except parotid secretions, were inhibitory. They suggested that complexes of the virus with high molecular weight submandibular mucins could play a role in viral inhibition [8]. A study by Wu *et al.*[9] and Malamud *et al. *[10] identified salivary agglutinin as a possible macromolecular component with a specific inhibitory role against HIV-1 through interaction with viral envelope protein gp120 and possible carbohydrate-mediated binding. The postulate of Archibald *et al.*[8] and the findings of Habte *et al.*[2,3] similarly suggest a specific interaction inhibitory of HIV-1, in this case by the high molecular weight mucin components of saliva, namely MUC5B and MUC7.

Mucins are high molecular weight glycoproteins with complex O-linked oligosaccharide side-chains that are found both in crude mucus gels and as transmembrane proteins on the apical cell surface**s** of glandular and ductal epithelia of various organs [11]. There are a whole host of transmembrane mucins with MUC1, MUC4 and MUC16, being very well characterized and shown to be aberrantly expressed in various malignancies including cystic fibrosis, asthma and cancer [12]. It is also known that oncogenic changes are associated with altered glycosylation patterns in mucins, generating novel and exploitable epitopes in the fight against many cancers [11]. Genetic polymorphism of MUC7 alleles has been shown to be associated with asthma in which a shorter MUC7 allele has protective properties [13] and a longer MUC2 allele may help protect atopic individuals [14]. MUC7 contains a central tandem repeat region and the most common allele is comprised of 6 tandem repeats each of 69 nucleotides (base pairs) (23 amino acids) [15]. The MUC5B gene is known to have a considerable polymorphism with respect to its 59-nucleotide tandem repeat region [16]. Desseyn* et al. *[16] observed 5 alleles in a sample population of 86 unrelated individuals due to 3–8 GC-rich direct repeats of 59 base pairs. Therefore, polymorphism in MUC genes is significant in its effect on the number of possible glycosylation sites and is likely to have an effect on the functional, physicochemical properties of mucin, and is relevant in the susceptibility and onset of certain diseases [15]. Indeed, the polymorphisms within MUC5B and MUC7 genes may play a role in the susceptibility to infection (by viruses such as HIV). It has been proposed that variable charge on the mucin molecule, due to altered glycosylation patterns may affect its viral binding properties and the agglutination of the viruses [2,17]. Nagashunmugam *et al.*[18] observed inter-individual variation, which they could quantify with respect to viral binding properties. There has been an increasing interest in mucins in the detection and treatment of carcinomas in general [19], in particular as diagnostic and therapeutic agents [20].

The work by Habte *et al.* in our laboratory on the role of mucus from HIV negative and HIV positive donors in its inhibition of HIV in saliva [2,3,21], breast milk [22,23] and cervical mucus [21], was an attempt to answer a novel question of the role of mucus and mucins in the inhibition of HIV-1. The limitation of that study [2] was that there was no proper control group because ‘normal’ was based on the declaration by the donor of the sample of having a ‘risk-free’ lifestyle. Also, the low yield of purified mucin from individual samples necessitated the pooling of such samples within both groups. This study which attempts to verify the findings of Habte *et al.*[2] has two well defined groups in which HIV negative samples were obtained from individuals tested for HIV-AIDS whilst HIV positive samples were obtained from patients clinically diagnosed with the disease.

## Results

### Gel filtration and separation of MUC5B and MUC7 in crude saliva

**Figure 1:**
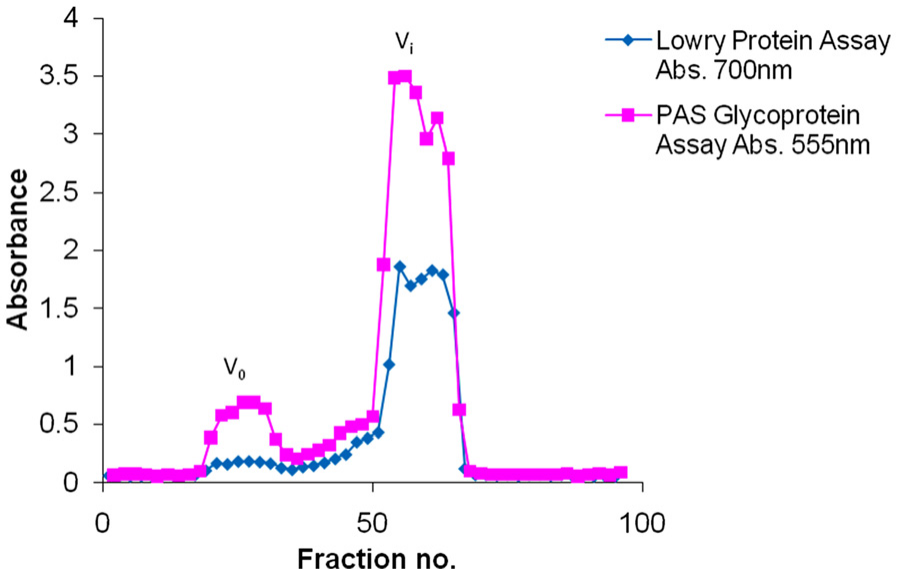
**The gel filtration profile of crude saliva from an HIV negative donor.** Crude saliva (20 ml in 6 M GuHCl, 10 mM EDTA, 1 mM PMSF pH 6.5) from an HIV negative donor was chromatographed on a Sepharose CL-4B gel filtration column (bed volume 200 ml). The column was eluted with 0.2 M NaCl: 0.02% NaN_3_ at a flow rate of 60 ml/h at room temperature. An aliquot of each fraction was assayed by the PAS and Lowry methods. V_0_ indicates the void volume and V_i_ indicates the included volume of the column. Fractions 18–34 were pooled to recover MUC5B and fractions 50–66 were pooled to recover MUC7. Fractions were dialysed against three changes of distilled water overnight at 4°C, and freeze-dried.

There was an inter-individual variation in the amounts of material eluting under the void volume (V_0_) and included volume (V_i_) peaks and the shape and size of the peaks, for both groups. The mucins in the void (V_0_) and included volumes (V_i_) of the column have previously been identified as MUC5B and MUC7 respectively [24]. In 3 of the 20 HIV negative and 5 of the 20 HIV positive samples respectively, the V_0_ peak was either absent or barely detectable whilst the V_i_ peak was seen for all samples. The V_0_ peak, when present was always smaller but slightly broader than the V_i_ peak which was sometimes split as shown in SDS-PAGE [3,21], suggesting the presence in some samples of two populations of MUC7, varying slightly in size. MUC7 also eluted with more protein positive material (proteins other than mucins) than MUC5B (V_0_) (Figure 1).

### Purification of mucin components by CsCl density-gradient ultracentrifugation

**Figure 2:**
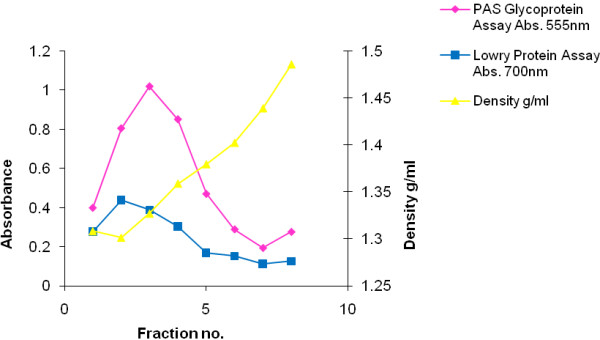
**Profile of purification by caesium chloride density-gradient ultracentrifugation of an HIV positive MUC7 sample.** Freeze dried material, in this example a sample of MUC7 (V_0_), was dissolved in 4 M GuHCl containing 10 mM EDTA, 5 mM NEM, and 0.05% CHAPS pH 6.5, and adjusted to a density of 1.39 to 1.40 g/ml with solid caesium chloride. Density-gradient centrifugation was performed in a Beckman L45 ultra-centrifuge for 48 h at 40 000 rpm/105 000 g, at 4°C. Fractions 3 and 4 were pooled, dialysed against 3 changes of distilled water overnight at 4°C and freeze-dried.

Mucins which eluted from the V_0_ and V_i_ peaks of the Sepharose gel filtration column were further purified by density gradient ultra-centrifugation in CsCl for 48 h at 105 000 g, twice. Mucin-rich material fractionated at the expected density of 1.42–1.45 g/ml, with most of the associated protein having been removed during the purification process. Apomucin (mucin protein backbone) is detected by the Lowry assay and seen as a smaller peak under the larger mucin peak (Figure 2). The mucin-rich fractions which eluted at densities close to 1.39–1.40 g/ml were pooled, dialysed and freeze dried for further analysis.

### Amino acid analysis

The average serine, threonine and proline (S, T, and P) content of the mucin for MUC5B was 27.47% and 28.55% for 3 HIV negative and positive samples respectively. MUC7 from HIV negative samples had an S, T, P content of 21.97% and that from HIV positive samples was 23.61%.

### Agarose gel-electrophoresis and Western blotting

**Figure 3:**
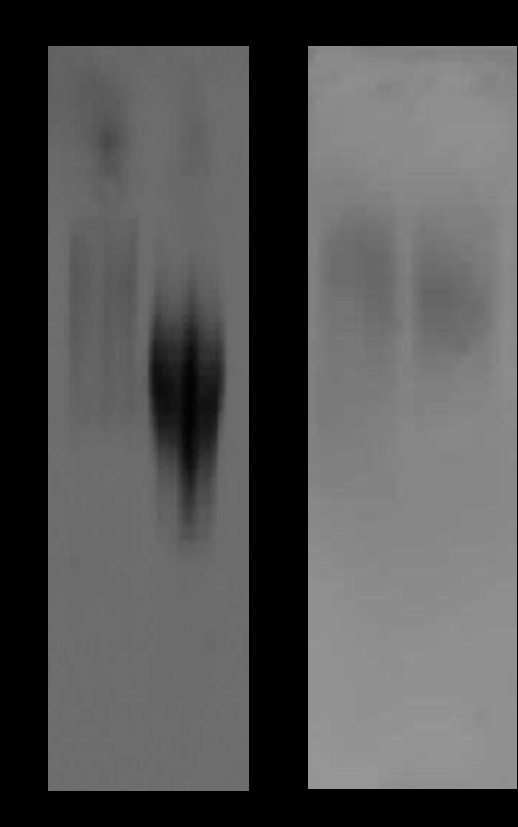
**Western blotting analysis (representative) of HIV negative and HIV positive salivary mucins MUC5B (a) and MUC7 (b).** a. lanes: 1, MUC5B (V_0_) HIV negative;2,MUC5B (V_0_) HIV positive. b. lanes: 1, MUC7 (Vi) HIV negative;2, MUC7 (Vi) HIV positive. Mucin proteins were detected using rabbit anti-MUC5B polyclonal and mouse anti-MUC7 monoclonal primary antibodies respectively. Dashed-arrows indicate loading position at the top of the gel picture and further arrows indicate the migration distance of samples.

Samples (n = 40) were run on 1% agarose gels and transferred onto nitrocellulose membrane by vacuum blotting. Mucin proteins were detected using rabbit anti-MUC5B polyclonal and mouse anti-MUC7 monoclonal primary antibodies respectively. Both the HIV negative and positive samples for material eluted from the V_0_ of the Sepharose 4B column (representation of samples shown in Figure 3a, lanes 1 and 2) showed a trace of MUC5B material near the top of the gel, with a smaller sized and more highly charged species visible further into the blot, that of the HIV positive sample being more intense and charged (Figure 3a, lane 2) than that of the control group (Figure 3a, lane 1). Migration of material is affected by the charge and the length of the oligosaccharide side-chains and the length of the polypeptide chain. Material eluted in the V_i_ of the Sepharose 4B column (Figure 3b, lanes 1 and 2) showed traces of MUC7 with similar intensities for the HIV negative (lane 1) and HIV positive (lane 2) samples.

### HIV-1 inhibition assay

On day 4 of the HIV-inhibition assay the HIV negative crude saliva group showed positivity for p24, an indication of viral infection, in all 20 samples from each group. Positivity for p24 in HIV positive crude saliva samples was significant with a ratio of 16/20 p = 0.0114. Four of twenty samples were negative for p24 in this group.On day 7, positivity for p24 in the HIV negative crude saliva group was 6/20. Fourteen samples showed negative results for p24, that is no viral infection occurred, in this group. Positivity for p24 in the HIV positive crude saliva group was 5/20. Fifteen of twenty samples were negative for p24 in this group. These results show a trend from day 4 to day 7 for a decrease in positivity for p24 for both HIV negative and HIV positive groups. Cross tabulation data shows that the p24 variable did not differ by HIV-status (Pearson Chi-square test shows no significant difference between groups p = 0.723, p > 0.05). The p24 was negative for 14/20 (70.0%) HIV negative crude saliva samples, and 15/20 (75.0%) HIV positive crude saliva samples. On day 7, 14/20 (70.0%) HIV negative samples were p24 negative, compared with HIV positive samples where 15/20 (75.0%) were p24 negative.

To compare the p24 antigen assay results from days 4 and 7 for each group (HIV negative and HIV positive crude saliva samples) McNemar statistical tests were performed. On day 4, all HIV negative samples gave a positive result for p24, hence no direct comparison between HIV negative and HIV positive groups was made at this stage. Comparing day 4 and day 7 p24 results for HIV positive samples a significant difference was found (p = 0.001, p < 0.05), therefore the negativity for p24 on day 7 was statistically significant.

Purified mucin samples of MUC5B and MUC7 for both HIV negative and HIV positive groups at dilutions of 10^-1^, 10^-3^, 10^-5^, 10^-10^, 10^-20^ and 10^-40^ were seen to inhibit HIV-1. The inhibition was observed for all but two MUC5B and two MUC7 samples tested from each group. Infection with HIV had occurred at one dilution of 10^-40^ for MUC5B indicating the potential for a dose–response relationship. Due to this small sample size a statistical analysis of this group was not possible.

### DNA analysis

**Figure 4:**
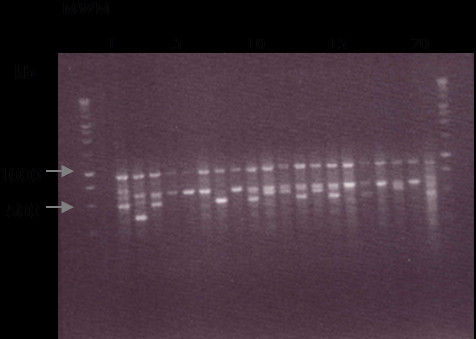
**Gel electrophoresis of the PCR product of MUC5B tandem repeat regions showing variations in the number of tandem repeats.** Gel electrophoresis of the PCR product of MUC5B tandem repeat regions showing variations in the number of tandem repeats. A 2% agarose gel was used for MUC5B DNA samples. 20ul of reaction mixture was loaded for each sample into each lane. MWM marks those lanes loaded with a DNA molecular weight marker. Ethidium bromide enabled DNA to be viewed under ultraviolet light. Lanes 1–10 are HIV negative DNA samples and 11–20 HIV positive DNA samples (a representation of samples is shown, lanes are numbered as such for clarity purposes).

Gel electrophoresis of MUC5B tandem repeat regions (Figure 4) revealed numerous polymorphisms between samples. There is some variation in the number of repeat polymorphisms in each of the samples from the HIV negative group compared with those in the HIV positive group*.* The sequence with 8 tandem repeats was the most common genotype in both groups. Analysis of heterozygosity of polymorphisms within MUC5B tandem repeat gene fragments for both populations revealed little difference between HIV negative and HIV positive groups. The HIV negative group was 53% heterozygous whilst the HIV positive group was 60% heterozygous (Figure 4).

**Figure 5:**
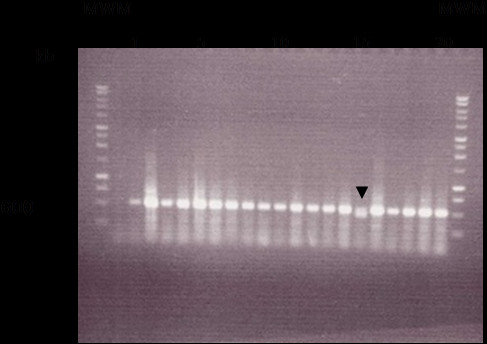
**Gel electrophoresis of the PCR product of MUC7 tandem repeat regions showing variations in the number of tandem repeats.** A 1% agarose gel was used for MUC7 DNA samples. 20ul of reaction mixture was loaded for each sample into each lane. MWM marks those lanes loaded with a DNA molecular weight marker. Ethidium bromide enabled DNA to be viewed under ultraviolet light. Lanes 1–10 are HIV negative DNA samples and 11–20 HIV positive DNA samples (a representation of samples is shown, lanes are numbered as such for clarity purposes). A single band (590 bp) represents the tandem repeat fragment with six repeats indicating a donor who is homozygous for MUC7. The double band, marked with an arrow, is of the fragment with six repeats and the smaller sized band (521 bp) is the fragment with five repeats. This indicates a heterozygous genotype for the donor for MUC7.

Gel electrophoresis of the tandem repeat regions of the MUC7 gene (Figure 5) revealed that the repeat structure for MUC7 was similar between all samples with no influence of HIV status. All patients were homozygous where the sequence contained 6 tandem repeats (590 bp) in each gene copy, except for one sample from the HIVnegative group that had a heterozygous genotype of a 6 tandem repeat and a 5 tandem repeat (521 bp) (Figure 5 arrow head).

## Discussion

This is the first study quantifying the novel observations made in our laboratory by Habte *et al. *[2] that crude saliva, from uninfected individuals and its mucins MUC5B and MUC7 inhibit HIV-1 in an *in vitro* assay. Crude saliva from HIV positive patients was not studied by Habte *et al.*[2,3,21]. The method we used was strictly according to that of Habte *et al. *[21], who did not test for mucin in the insoluble debris. This debris was shown to have some mucin in saliva preparations, which was not added to that in the supernatant in this study. Any HIV particles in the pellet would be rendered denatured by the 6 M guanidinium hydrochloride in the extraction media. Besides confirming Habte’s finding on normal saliva and salivary mucin [2], we have gone on to show that crude saliva and its purified mucins from HIV infected individuals also inhibited HIV-1, in contrast to the findings of Habte *et al.*[2,3,21], who reported that HIV positive mucins did not inhibit the virus.

We consider this study to be quantitative in the sense that it differs from previous studies of Habte *et al. *[2] in that there were two major groups in this study, a diagnostically confirmed normal (n = 20) and infected group (n = 20), and there was no pooling of sample in either group. We do not claim to quantify mucins strictly by assay because of the difficulties such a measurement poses, due to the polydisperse or even heterogenous nature of crude mucus secretions.

This quantitative study had several advantages over the previous qualitative study of Habte *et al. *[2], in our laboratory. Firstly, the HIV negative group of twenty individuals were tested for HIV, unlike previously where samples were taken on the basis of trust from patients declaring a risk-free lifestyle with respect to sexual habits and other risky behaviours such as the abuse of drugs. Furthermore this study compared the inhibitory potential of crude saliva in both groups together with that of the mucins MUC5B and MUC7. Since Habte *et al. *[2] did not test crude saliva from HIV positive individuals and focussed only the salivary mucins MUC5B and MUC7 purified from a pool of HIV positive saliva [3], our findings raise new questions, encouraging us to design future studies that will take into account the treatment status of patients, the extent of the infection in relation to the CD4 counts and a wider genetic study of mucins in a population in this region. It is interesting that a few samples in both groups did not inhibit the virus. This may be due to inter-individual variation in mucin concentration of the crude saliva samples. The establishment of a dose–response curve, which we are busy with, will help answer such questions. It had not been done because of limitations in terms of time constraints, sample yield (we used individual samples) and location (these experiments were carried out in the Division of Virology at the University of Stellenbosch in which there was this established assay made available to us and in which laboratory space was shared due to restricted conditions).

Crude saliva is conveniently separated into its mucin components with MUC5B eluting in the V_0_ and MUC7 in the V_i_ of a Sepharose CL-4B gel filtration column. However, there was inter-individual variation within normal and infected groups, especially for MUC5B which in some instances seemed hardly detectable, whilst in other cases eluted as a small but broad peak from the column. When comparing the biochemical properties of mucins from normal saliva and that from HIV-infected patients, both groups had more MUC7 material than MUC5B. The reason for this is unknown but it is not an exact measure in the strict sense of a mucin assay (with reference to a standard curve), but rather a more general assessment based on the size of peaks eluting from the column, an indicator of amounts of material on a broad comparative basis, like has been previously reported in other studies on mucins in the stomach [25,26]. All samples showed a larger MUC7 peak, the shape of which differed from sample to sample and was associated with considerable amounts of protein compared to the MUC5B of the void volume. In some instances this peak was split, suggesting a variation in the MUC7 population, in keeping with Habte’s findings of two bands of purified MUC7 on Western blotting after 4-20% gradient gel electrophoresis [3].

Crude saliva and purified mucins from both groups inhibited the virus in an *in vitro* assay. Mucins were purified by density gradient ultra-centrifugation in CsCl, a long-established procedure shown to free mucins in complex secretions from contaminating protein [27-29]. Salivary mucins MUC5B and MUC7, purified from the saliva of HIV positive patients, were previously shown by 4-20% gradient SDS-PAGE to be pure and Western Blotting confirmed the identity of these mucins [3]. The amino acid analysis is characteristic of purified O-glycosylated mucin in its serine, threonine and proline (S, T and P) content [29], which was slightly higher in the HIV positive group. The significance of this is not known and since only 3 samples from each group were analysed, a statistical analysis was not done.

It may be worthwhile to determine further whether the roles of MUC5B and MUC7 vary in the inhibition of HIV-1. A study by Thomsson *et al *[30] highlights the differences in glycosylation between MG1 (MUC5B) and MG2 (MUC7). They identified that MG1-derived oligosaccharides (sugar side chains attached to the protein molecule) were significantly longer than those of MG2. This is in keeping with the findings in this study that MUC5B was a larger molecule than MUC7 and eluted in the void volume (V_0_) of the Sepharose Cl-4B column, although the length and size of the polypeptide may not necessarily relate to the length of oligosaccharides. A greater extent of glycosylation may be indicative of a more relevant role in the inhibition of HIV-1 transmission. Interestingly it is a feature of cancer-related mucins to be under-glycosylated and to have an altered glycosylation pattern when compared with normal mucins [11,31]. A more glycosylated molecule with longer oligosaccharides could more effectively aggregate virus particles. Some of these questions would form the basis for future research.

Western blotting detection of purified salivary mucin samples revealed a large amount of MUC5B in HIV negative samples compared with trace amounts of MUC5B in HIV positive samples. MUC7 was detected in both HIV negative and HIV positive samples (Figure 3b). Here however the charge of the oligosaccharides on the apomucin can influence migration of the mucin through an agarose gel.

Purified salivary mucins MUC5B and MUC7 with dilutions of 10^-1^, 10^-3^, 10^-5^, 10^-10^, 10^-20^ and 10^-40^, provided a wider range than that used by Habte *et al. *[2], and showed the potency of the mucin in the inhibition of the virus at very low concentrations and high dilutions. The finding of Habte *et al.*[3] that MUC5B and MUC7 from the saliva of patients who are HIV positive did not inhibit mucins is an essential difference to our finding in this study. If any variation existed in the inhibitory potential of mucins from sample-to-sample, this would be masked by the pooling of these individual samples. There is a large amount of other protein contaminants in crude saliva and their inhibitory potential needs to be investigated. These include cystatins (inhibitors of cysteine proteases), antibodies (sIgA) and, in addition to mucins, other high molecular weight glycoproteins such as salivary agglutinin (SAG) [9,32]. SAG has been shown to have a specific inhibitory effect through interaction with viral capsid glycoprotein gp120 [9]. A larger study is being planned to investigate these questions, especially a comparison of the inhibitory properties of mucins versus other components such as gp340 in the inhibition of the virus.

DNA analysis of tandem repeat regions in the genes of MUC5B and MUC7 from HIV negative and HIV positive donors revealed no association of HIV-infection status and gene polymorphisms. Polymorphisms are distributed between both groups. There is no apparent link between heterozygosity or homozygosity in either the MUC5B or MUC7 tandem repeat alleles and HIV-infection. This suggests that there is no genetic predisposition for the susceptibility to HIV-infection. An investigation of the variation in the genetic and protein structure of mucins MUC5B and MUC7 and their association with infection with HIV is a study that could be engendered by these findings.

One area of research aimed at the reduction of infection in this region is the development of microbicides which can enhance the body’s first line of defence against the virus. A formulation or preparation, in which mucins could form a part of a barrier substance, may be an idea worth exploring. Secreted mucins such as MUC5B form gels in the respiratory and other internal tracts of the body which protect epithelia lining the mucosae of these tracts from harmful factors in a hostile milieu. One such example is the crude mucus gel lining the surface of the gastric mucosa, a 200 μM thick barrier which forms an unstirred layer on the gastric mucosal surface and protests it from hydrochloric acid (down to pH 1), pepsin activity and the shear forces associated with digestion [33]. Our findings suggest that the virus could be trapped by mucins in the saliva. We suggest that the biochemical structure of these mucins, together with the physical properties of the crude mucus, could make a mucus-based formulation (a substance or preparation that contains mucins or even crude mucus as a component of the preparation) an effective barrier, protecting the vaginal mucosa against the shear associated with sexual intercourse and remaining intact long enough to resist the infection of the mucosal cells by the HI virus. Studies amenable to identifying a peptide or short polypeptide with activity that could be developed into a microbicide are also in progress.

## Conclusions

Crude salivary mucus and the purified salivary mucins MUC5B and MUC7 play a role in the inhibition of HIV-1 in the oral cavity. This inhibition is not limited to saliva and its purified mucins from HIV negative donors, but includes that from HIV positive patients.

## Methods

### Ethics

The University of Cape Town Research and Ethics Committee approved this study (ethics approval number REC REF: 283/2004). Prior to collection of sample, each patient or participant in the study was provided with a consent form and an information sheet explaining the nature and scope of the project. Informed consent was then obtained by signature of the patient/participant, before the sample was collected.

### Saliva collection, mucin preparation and analysis

Saliva was collected from tested HIV negative individuals (n = 20) (Voluntary Counseling and Testing Drive at the University of Cape Town) and HIV positive patients (n = 20) (HIV-positive Clinic, Groote Schuur Hospital, Cape Town). The preparation and purification of salivary mucins was according to the method described by Habte *et al.*[2]. Briefly, each sample of crude saliva (supernatant fluid after gentle stirring and centrifugation to remove large debris) was separated by Sepharose CL-4B and subsequently purified by caesium chloride isopycnic density gradient ultra-centrifugation [34]. Mucins were further analysed by agarose gel electrophoresis and Western blotting [35]. Amino acid analysis was performed for 3 samples from each group [35]. Glycoprotein was estimated by the PAS procedure of Mantle and Allen [36] and protein according to the method of Lowry *et al.*[37].

### HIV-1 inhibition assay

The inhibition assay was a variation of the one described by Habte et *al.*[2,21]. It allowed for the detection of the anti-HIV-1 activities of crude saliva and purified salivary mucins MUC5B and MUC7 from both HIV negative and HIV positive individuals in *in vitro* cell culture experiments as described by Nagashunmugam *et al.*[18]. Mucin at a concentration of 1 mg/ml was dissolved in 0.25% PBS (dilution in distilled water) and a dose–response curve established using dilutions of 10^-1^, 10^-3^, 10^-5^, 10^-10^, 10^-20^ and 10^-40^ of the original concentration. HIV-1 plus media (RPMI 1640 with 10% fetal calf serum, penicillin and streptomycin, obtained from Gibco, Massachusetts, USA) was used as a positive control. Negative controls used were culture media alone and media incubated with PBMCs (Peripheral blood mononuclear cells).

The virus used in the assay was TV167, a subtype C HIV-1 isolate that is the most common subtype in Southern Africa. This specific isolate is a dualtropic strain that expresses CCR5 and CXCR4 co-receptors equally, mimicking the virus during all stages of its proliferation during an infection and therefore reducing variables for the experiment. Another subtype C HIV-1 isolate (TV671) was included in the experiments as an additional positive control.

One hundred microlitres of pure mucin sample was mixed with 400ul HIV-1 supernatant fluid (p24 antigen-positive supernatant after virus isolation by PBMC co-culture) and incubated for 60 min at 37°C (5% humidity, 95% [CO_2_]). The samples were then filtered using 1.0 ml 0.25% PBS through a 0.45um hydrophilic cellulose acetate membrane (PRO-X Filter Unit, Lida Manufacturing Corp.). After incubation and filtration, the filtrate containing free virus was discarded, and the unfiltered (retained) samples (containing mucin components and ‘trapped’ virus particles) were recovered using 0.5 ml 0.25% PBS.

PBMCs were isolated using the Ficoll density-gradient method after being pooled from different HIV negative donors and stimulated with PHA (4 days before the assay) and IL −2 (two days before the assay). Four hundred microlitres of the unfiltered sample was then incubated with 100ul of PBMCs at 37°C (5% humidity, 95% [CO_2_]), the plate centrifuged on day 4, at 1000 rpm for 10 min and the supernatant fluid harvested and stored at −80°C for the p24 ELISA assay (Vironostika HIV-1 Antigen kit was obtained from Biomerieux, Netherlands). Media was replaced with cells and cultured and viral infection of the cells was measured using an assay for the quantitative estimation of p24 antigen. The qualitative cut off value was calculated by the average optical density values of the 3 p24-negative kit controls plus a constant of 0.07. Samples with absorbance values less than the cut-off value are considered nonreactive by the criteria of Vironostika HIV-1 antigen kit (Biomerieux, Netherlands) and may be considered negative for HIV-1 antigen.

### DNA extraction and analysis

DNA analysis was performed in order to establish whether there is a difference between HIV negative and HIV positive samples in the tandem repeat region of the mucin genes MUC5B and MUC7. The DNA was extracted and purified from buccal swabs taken from HIV negative (n = 20) and HIV positive (n = 20) saliva donors. The swabs were allowed to air dry and centrifugation steps were carried out at RT prior to DNA extraction procedures. The protocol for DNA purification from buccal swabs (Spin protocol) for isolation of total genomic, mitochondrial and viral DNA and RNA was followed as described in the QIAamp DNA Mini and Blood Mini Handbook and used a QIAamp DNA Mini kit (obtained from Southern Cross Biotechnology). PCR (polymerase chain reaction), of the tandem repeat regions of each of the MUC genes allowed for polymorphisms in these regions to be identified and any correlation with the HIV infection status of the donor to be noted.

### Gene amplification using PCR

PCR was used to determine the presence or absence of polymorphisms in the tandem repeat regions of MUC5B and MUC7. Primers flanking these tandem repeat regions were used to amplify samples from both HIV negative and HIV positive individuals. PCR primers (Whitehead Scientific):MUC7 – Primer 1 (forward): 5’-GTA GCT ACA TTA GCA CCA GTG-3’; primer 2 (reverse): 5’-TTC AGA AGT GTC AGG TGC AAG-3’.MUC5B – Primer 1 (forward): 5’-AGT GTG CAG TGA CTG GCG AG-3’;primer 2 (reverse): 5’-CTA GAG TTG CAG GTG GCA GG-3’.

Each PCR consisted of a 50ul reaction containing of 1X GoTaq buffer (Promega, Wisconsin), 3 mM MgCl, 200uM of each dNTPs, 1uM of each primer, approximately 0.5ug of DNA and 2.5 units of GoTaq DNA polymerase (Promega, Madison, Wisconsin). Amplification conditions for MUC5B were as follows: initial denaturation at 95°C for 3 min followed by 30 cycles of denaturation at 95°C for 30secs, primer annealing at 60°C for 30secs and extension at 72°C for 45secs. A final extension step was done at 72°C for 7 min. Amplification conditions for MUC7 were as follows: initial denaturation at 94°C for 2 min and then 30 cycles of denaturation at 94°C for 30secs, primer annealing at 60°C for 30secs and extension at 72°C for 30secs. A final extension step was done at 72°C for 6 min.

### Agarose gel electrophoresis for DNA polymorphism analysis

MUC5B and MUC7 products were analysed on a 2% and 1% agarose gel respectively. Twenty microlitres of reaction mixture was loaded for each sample and a 1 kb DNA ladder (Promega, Wisconsin) was used to determine the sizes of bands. Gels were run in 1X TAE (Tris acetic acid with EDTA) buffer at 60 V for 2 h. Ethidium bromide (EtBr) enabled the DNA to be viewed using ultraviolet light.

### Statistical analysis of data

Under the hypothesis that 70% of patient samples in the two compared groups (HIV negative and HIV positive samples) would be p24-positive in the HIV-1 inhibition assay, and based on a power of 0.80 and a level of significance of 0.05, an estimated sample size of 18 patients was calculated. Thus, a sample size of 20 subjects was selected for this study.

Categorical data was compared using Pearson Chi-square tests (or the Fisher’s exact test) whenever appropriate. The McNemar test was used for comparing the p24 assay results at days 4 and 7 of the HIV-1 inhibition assay. All tests were 2-sided and a p-value <0.05 was considered as significant.

## Abbreviations

HIV-1 = Human Immunodeficiency Virus Type 1; PBMC = Peripheral blood mononuclear cell; CsCl = Caesium chloride; STP = Serine, Threonine, Proline; GuHCl = Guanidinium hydrochloride; EDTA = Ethylenediaminetetra-acetic acid; PMSF = Phenylmethylsulfonylfluoride; PAS = Periodic acid Schiff base; NaCl = Sodium chloride; NaN3 = Sodium azide; RT = Room temperature, approximately 25°C; SDS-PAGE = Sodium dodecyl sulphate – Polyacrylamide gel electrophoresis; TBST = Tris buffered saline with 0.05% Tween-20; HRPO = Horse radish peroxidase; HPLC = High performance liquid chromatography; PBS = Phosphate-buffered saline; PHA = Phytohemagglutinin; IL-2 = Interleukin-2; ELISA = Enzyme-linked immunosorbent assay; PCR = Polymerase chain reaction; MgCl = Magnesium chloride; dNTP = Deoxynucleotide triphosphates; kb = Kilobase; MWM = Molecular weight marker; TAE = Tris acetic acid with EDTA; EtBr = Ethidium bromide; SAG = Salivary agglutinin.

## Competing interests

The authors declare that there are no competing interests.

## Authors’ contributions

JP is reading for an MSc degree and this work is her topic. She prepared the first draft of the manuscript and saw it to completion. ZL helped with the biochemistry experiments. CdB established and oversaw the *in vitro* assay. PR enabled the recruitment of HIV-positive donors. ASM originated the idea, supervised the project, and edited the manuscript. All authors read and approved the final manuscript.

## Authors’ information

JP is reading for her MSc in Surgical Sciences. ZL is a medical technologist and research assistant. CdB is a virologist. PR is an HIV clinician. ASM is a biochemist and the Manager of the Research laboratory.
